# ConsensuSV—from the whole-genome sequencing data to the complete variant list

**DOI:** 10.1093/bioinformatics/btac709

**Published:** 2022-10-31

**Authors:** Mateusz Chiliński, Dariusz Plewczynski

**Affiliations:** Laboratory of Bioinformatics and Computational Genomics, Faculty of Mathematics and Information Science, Warsaw University of Technology, Warsaw 00-662, Poland; Laboratory of Functional and Structural Genomics, Centre of New Technologies, University of Warsaw, Warsaw 02-097, Poland; Laboratory of Bioinformatics and Computational Genomics, Faculty of Mathematics and Information Science, Warsaw University of Technology, Warsaw 00-662, Poland; Laboratory of Functional and Structural Genomics, Centre of New Technologies, University of Warsaw, Warsaw 02-097, Poland

## Abstract

**Summary:**

The detection of the structural variants (SVs) using Illumina sequencing of human DNA is not an easy task. Multiple approaches have been proposed; however, all the methods have their limitations. In this article, we present ConsensuSV pipeline that aids the research in complex variant detection. By using consensus meta-approach, eight independent SV callers are being used to identify a uniform set of high-quality SVs. The pipeline works using raw sequencing data and performs all the necessary steps automatically, significantly reducing the researchers’ time required for processing the data. The output files contain SVs, single nucleotide polymorphisms and Indels. The pipeline uses *luigi* framework, allowing the software to be run efficiently and parallelly using the high-performance computing infrastructure. We strongly believe that the software is useful to the scientific community interested in the germline variant detection.

**Availability and implementation:**

https://github.com/SFGLab/ConsensuSV-pipeline.

**Supplementary information:**

Supplementary data are available at *Bioinformatics* online.

## 1 Introduction

The structural variants (SVs) recently have risen to the forefront of the computational biology and medical genomics research focus. The advancement of methods available to the researchers leads to more sensitive detection, allowing the functional study of complex genomic diseases caused by the rare SVs [reviewed in (Chiliński *et al.*, 2021)].

Multiple SV callers have been created to detect the variants from the whole-genome sequence. However, as they implement different approaches, their results vary in terms of quality and quantity. Especially, using short-read data is error-prone—as the SVs contribute up to 3 Mbps difference from the reference human genome (Feuk *et al.*, 2006); therefore, the need emerged for providing optimized consensus out of multiple SV-detecting tools. As each of the SV callers implement different algorithms for detection, generating consensus out of them will ensure the high quality of the variants for improved functional analysis.

The median number of SVs in the human genome was estimated in 2015 (Sudmant *et al.*, 2015) to be approximately equal to 4405. However, that data were obtained using short-read Illumina data with mean 7.4-fold coverage. The current findings (Chaisson *et al.*, 2019) show, that this number is actually much higher. The authors use short-read Illumina data, along with long-read PacBio and Bionano Genomics technologies. The average total DNA sequence coverage of their data is 223.56. The results of mixing those experimental methods result in the discovery of on average 27 622 SVs per individual, a 7-fold increase from 2015 (Chaisson *et al.*, 2019).

There are various algorithms for the detection of SVs (especially in the case of the short-read data) and constructing the consensus out of them is starting to be the common strategy. Multiple tools have been proposed, and currently, the state-of-the-art tools are MetaSV (Mohiyuddin *et al.*, 2015) and FusorSV (Becker *et al.*, 2018); however, some of them lack flexibility (e.g. they have fixed SV callers that can be used for consensus extraction), others are flexible but lack the recently introduced advanced methods that would improve the overall quality. In this article, we introduce our own meta-caller, alongside with fully automated pipeline for the variant discovery that resolves all the issues we have encountered while using single tools, or meta-algorithms.

## 2 Implementation

The implementation of the software is divided into two modules—first one, *ConsensuSV-core*, is for getting the consensus out of the already-called SVs, which takes as an input VCF (Variant Call Format) files, and return merged meta-VCF file. The second module is a wrapper for the first one, called *ConsensuSV-pipeline*, which is the complete out-of-the-box solution capable of calling all types of variants, using as the input raw Illumina fastq files, and returning as the output the complete lists of SVs, Indels and SNPs (Single Nucleotide Polymorphisms) with minimal user involvement.

### 2.1 ConsensuSV-core

The *ConsensuSV-core* algorithm uses the calls from the individual SV identification algorithms. ConsensuSV starts by pre-processing all the individual VCF files to establish a unified format for further processing. Next, every SV from any of the tools is loaded into memory and iterated to find the list of close ones in terms of their starting position, ending position and type. If the minimum requirement (set by a user) of the number of overlapping candidates is reached, the tool continues processing the list of variants. The meta-tool checks if most of the candidates are the same—if they are, then any of them is selected and the consensus is created based upon it.

A problem arises when there are no majority candidates, yet at the same time being similar to each other. In this case, the pre-trained neural network model (see Supplementary material) is used for the generation of consensus breakpoints positions. Moreover, our meta-tool allows preparing a new machine learning model using the user-provided true breakpoints locations, which can be used to train the network. Having established consensus, the tool uses a simple voting algorithm among genotype information provided by individual tools to decide upon the genotype.

**Fig. 1.**
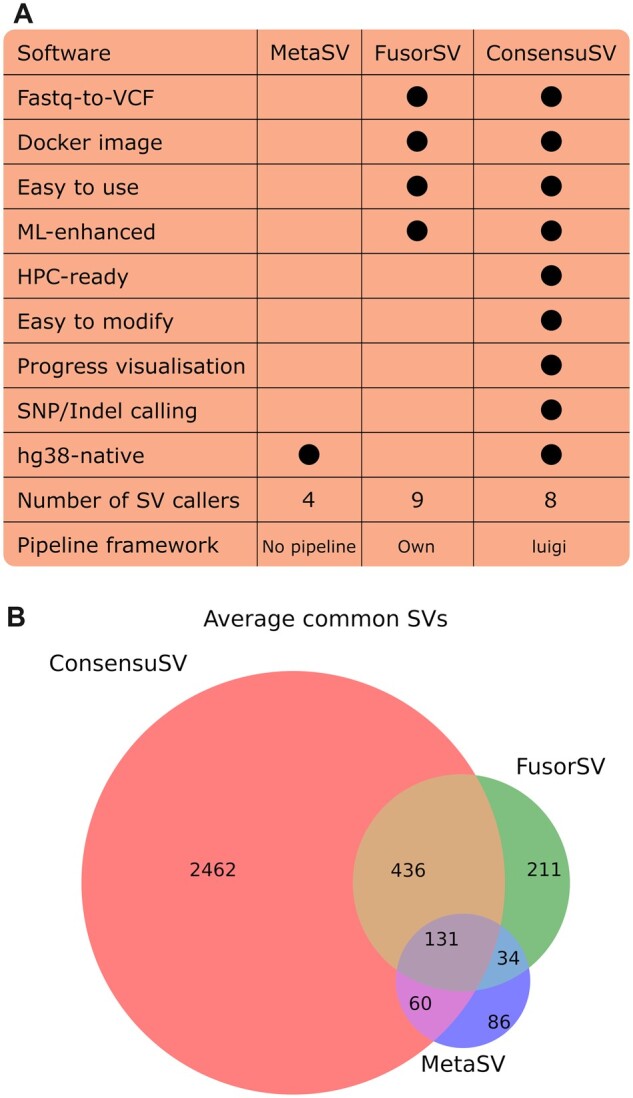
(**A**) Architectural differences between ConsensuSV, FusorSV and MetaSV. (**B**) Comparison of SVs (breakpoints) detected by ConsensuSV, FusorSV and MetaSV

We have compared our software to the before mentioned state-of-the-art callers, MetaSV and FusorSV. All the callings and comparisons were done using nine samples from NYGC (New York Genome Center) (Byrska-Bishop *et al.*, 2022), and we compared the breakpoints only (see Supplementary material). On average, we are reporting around 61% of the SVs that are also found by MetaSV, and around 70% of the ones from FusorSV (Fig. 1B). We have also compared those meta-callers with the gold-standard Illumina callset (Chaisson *et al.*, 2019), which we considered to be the ground truth for the comparison purposes. In that scenario, our software outperformed the other meta-callers in terms of precision and reached 4-fold increase in recall comparison to FusorSV, and 17-fold to MetaSV (see Supplementary material).

### 2.2 ConsensuSV-pipeline

The ConsensuSV-pipeline is based on the *luigi* framework and takes care of running the specific tasks in the appropriate order, visualization and control of the status for the particular tasks. The pipeline starts with the raw fastq files from Illumina sequencing, performs all the necessary preprocessing steps and then uses eight SV callers and merges them using *ConsensuSV-core*. The software is much easier to use than its competitors—FusorSV and MetaSV (Fig. 1A).

## 3 Discussion

In this article, we propose ConsensuSV as a complete out-of-the-box solution for the variant discovery, allowing a user to process the whole-genome sequencing data. The ease of the use and modularity of the framework allows many extensions, including adding novel algorithms for the SV detections, and the further improvement of the meta-caller using deep learning statistical learning model trained on multiple experimental technologies.

## Funding

This work was supported by National Science Centre, Poland (2019/35/O/ST6/02484 and 2020/37/B/NZ2/03757), and European Commission Horizon 2020 Marie Skłodowska-Curie ITN Enhpathy grant ‘Molecular Basis of Human enhanceropathies’. Research was co-funded by Warsaw University of Technology within the Excellence Initiative: Research University (IDUB) programme. Computations were performed thanks to the Laboratory of Bioinformatics and Computational Genomics, Faculty of Mathematics and Information Science, Warsaw University of Technology using the Artificial Intelligence HPC platform financed by Polish Ministry of Science and Higher Education [decision no. 7054/IA/SP/2020 of 2020-08-28].

*Conflict of Interest*: none declared.

## Supplementary Material

btac709_Supplementary_Data
