# Supplementary material for: ConsensuSV—from the whole-genome sequencing data to the complete variant list
Source: Bioinformatics. 2022 Oct 31;38(24):5440–2. doi: 10.1093/bioinformatics/btac709 (PMC9750118; doi:10.1093/bioinformatics/btac709)
Supplement: btac709_Supplementary_Data [file btac709_supplementary_data.zip › btac709_Supplementary_Data/Supplementary.docx]

Supplementary information

Table of Contents

[Tools used by the pipeline 2](#_Toc117005746)

[Structural Variant callers 2](#_Toc117005747)

[Actively used software 2](#_Toc117005748)

[ConsensuSV-core algorithm 3](#_Toc117005749)

[Finding similar (candidate) SVs between tools 3](#_Toc117005750)

[Deep learning model of breakpoint positions 6](#_Toc117005751)

[Parameters of minimal overlap 7](#_Toc117005752)

[Genotyping 8](#_Toc117005753)

[Meta-caller analysis 8](#_Toc117005754)

[ConsensuSV-pipeline 9](#_Toc117005755)

[MetaSV 9](#_Toc117005756)

[FusorSV 11](#_Toc117005757)

[Benchmark study 12](#_Toc117005758)

[Comparisons using SVBench 12](#_Toc117005759)

[Comparisons using Venn diagrams 13](#_Toc117005760)

[References 14](#_Toc117005761)

# Tools used by the pipeline

## Structural Variant callers

We have used 8 gold-standard tools for obtaining the consensus - BreakSeq2 (Abyzov *et al.*, 2015), BreakDancer (Chen *et al.*, 2009), CNVNator (Abyzov *et al.*, 2011), Delly (Rausch *et al.*, 2012), Lumpy (Layer *et al.*, 2014), Manta (Chen *et al.*, 2016), Tardis (Soylev *et al.*, 2017), and Whamg (Kronenberg *et al.*, 2015). Lumpy, Manta, Whamg, and Delly were chosen as they have been selected as one of the best ones to detect various structural variant types - namely deletions, duplications, insertions, and inversions (Kosugi *et al.*, 2019). Tardis has been used for his ability to detect complex SV (Soylev *et al.*, 2019), which it has been designed for. We considered those mutations to be a great addition to the output set detected by our tool. BreakSeq2 was used as a highest-precision tool - as it aligns to already well-established SV breakpoints (Abyzov *et al.*, 2015). BreakDancer and CNVNator were chosen as they are well-established tools for SV discovery, used in e.g. 1000 Genomes Phase 3 (Auton *et al.*, 2015). The summary of algorithms and outputs used by each of those tools is presented in the **Supplementary Table 1**.

| SV caller | Method | DEL | INS | DUP | INV | TRA |
| --- | --- | --- | --- | --- | --- | --- |
| BreakSeq2 | Alignment to known SV breakpoints | X | X |  |  |  |
| BreakDancer | RP | X | X |  | X | X |
| CNVNator | RD | X |  | X |  |  |
| Delly | RP+SR | X | X | X | X | X |
| Lumpy | RP+SR+RD | X |  | X | X | X |
| Manta | RP+SR+LA | X | X | X | X | X |
| Tardis | RP+SR+RD | X | X | X | X | X |
| Whamg | RP+SR | X | X | X | X |  |
| Total |  | 8 | 6 | 6 | 6 | 5 |

**Supplementary Table 1.** Summary of algorithms used in ConsensuSV-Pipeline metacaller.

## Actively used software

The tools that are actively used by the pipeline are presented in the **Supplementary Table 2**.

| **Software** | **Link** |
| --- | --- |
| anaconda | <https://www.anaconda.com/> |
| htslib | <https://github.com/samtools/htslib> |
| samtools | <https://github.com/samtools/samtools> |
| bcftools | <https://github.com/samtools/bcftools> |
| bwa | <https://github.com/lh3/bwa> |
| bwakit | <https://github.com/lh3/bwa/tree/master/bwakit> |
| biobambam2 | <https://github.com/gt1/biobambam2> |
| root | <https://root.cern/> |
| GATK | <https://gatk.broadinstitute.org/hc/en-us> |
| FastQC | <https://www.bioinformatics.babraham.ac.uk/projects/fastqc/> |
| vcftools | <http://vcftools.sourceforge.net/> |
| CNVNator | <https://github.com/abyzovlab/CNVnator> |
| breakdancer | <https://github.com/genome/breakdancer> |
| delly | <https://github.com/dellytools/delly> |
| breakseq | <http://bioinform.github.io/breakseq2/> |
| manta | <https://github.com/Illumina/manta> |
| tardis | <https://github.com/BilkentCompGen/tardis> |
| svelter | <https://github.com/mills-lab/svelter> |
| wham | <https://github.com/zeeev/wham> |
| ConsensuSV | <https://github.com/SFGLab/ConsensuSV> |

**Supplementary Table 2.** Actively used software in ConsensuSV-Pipeline.

# ConsensuSV-core algorithm

## Finding similar (candidate) SVs between tools

Finding of the similar (candidate) SVs works in iterative way - for each of the SVs from the first tool, we are looking for the SVs that are in close proximity. By SVs in close proximity, we consider the ones that start and end in similar positions. To ensure we detect all of them, we represent beginning and end of each SVs as intervals:

Beginning: *(pos+cipos1, pos+cipos2)*

End: *(end+ciend1, end+ciend2)*

The beginning interval of the SV1 must overlap the beginning interval of SV2, and the same for the ending. Additionally, there is allowed mismatch of the intervals by 100 base pairs. The concept of one single comparison is presented in the **Supplementary Figure 1**.


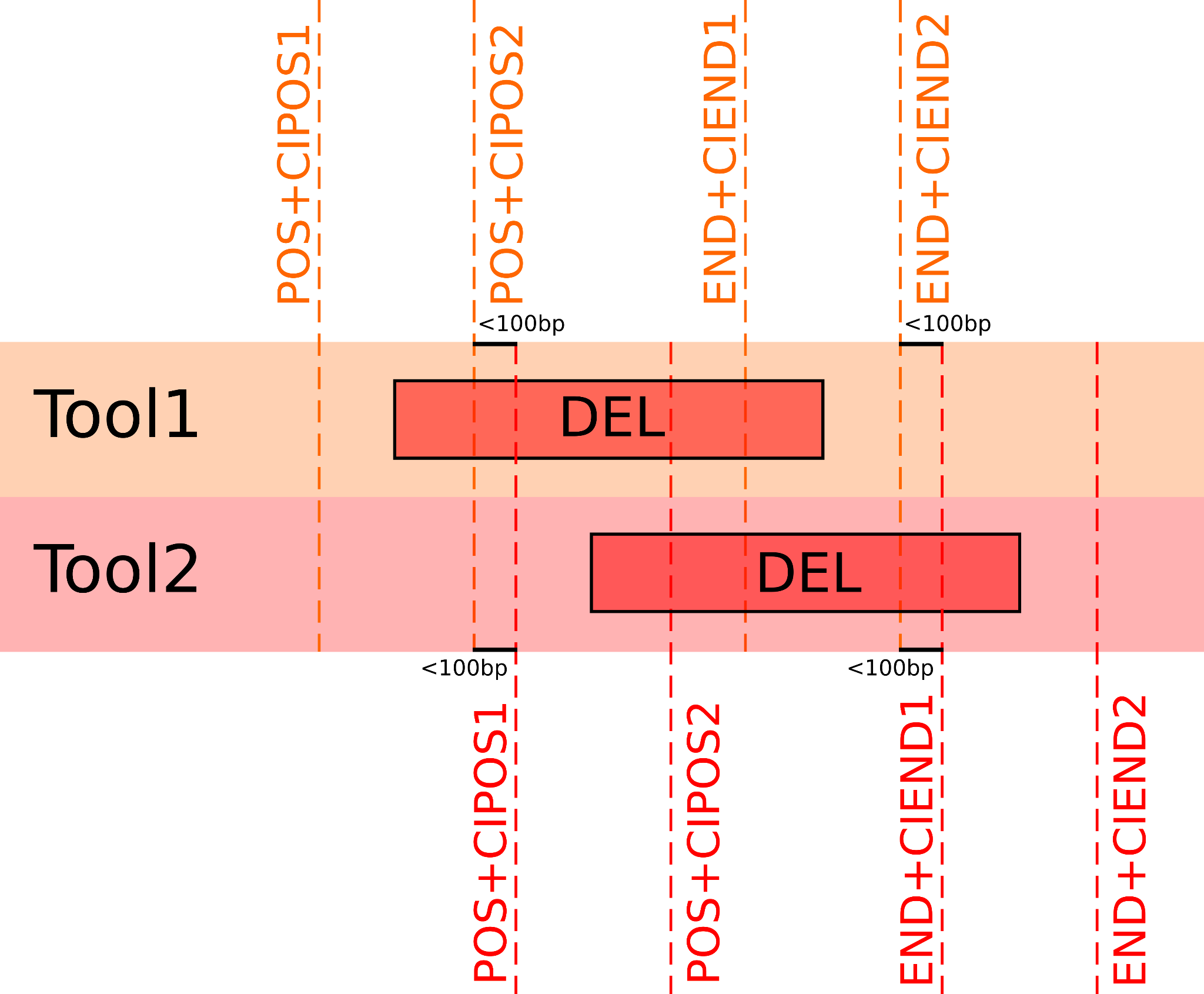


**Supplementary Figure 1.** Finding candidate SVs – checking, if two SVs overlap.

We go one-by-one and compare SVs from the first tool, with all the SVs in the other tools. If we find the overlap that will be used for creating consensus, we remove all the SVs that will be used for that establishment from the remaining set of SVs to process. Once we finish processing the first tool, we proceed to the next ones. That way, all the SVs are evaluated, and the ones that are already being used for the consensus are not evaluated again (since they are removed from the remaining set).

The output from this procedure gives us set, where each element contains multiple candidate SVs, that will be used for the consensus establishment. If all the candidates in one set element are exactly the same, we simply create consensus based on that. If not, we are using our deep learning model for the creation of the consensus SV. The whole workflow of the algorithm is presented in the **Supplementary Figure 2**, and the pipeline wrapping ConsensuSV-core into fully automated package (ConsensuSV-pipeline) is shown in the **Supplementary Figure 3**.


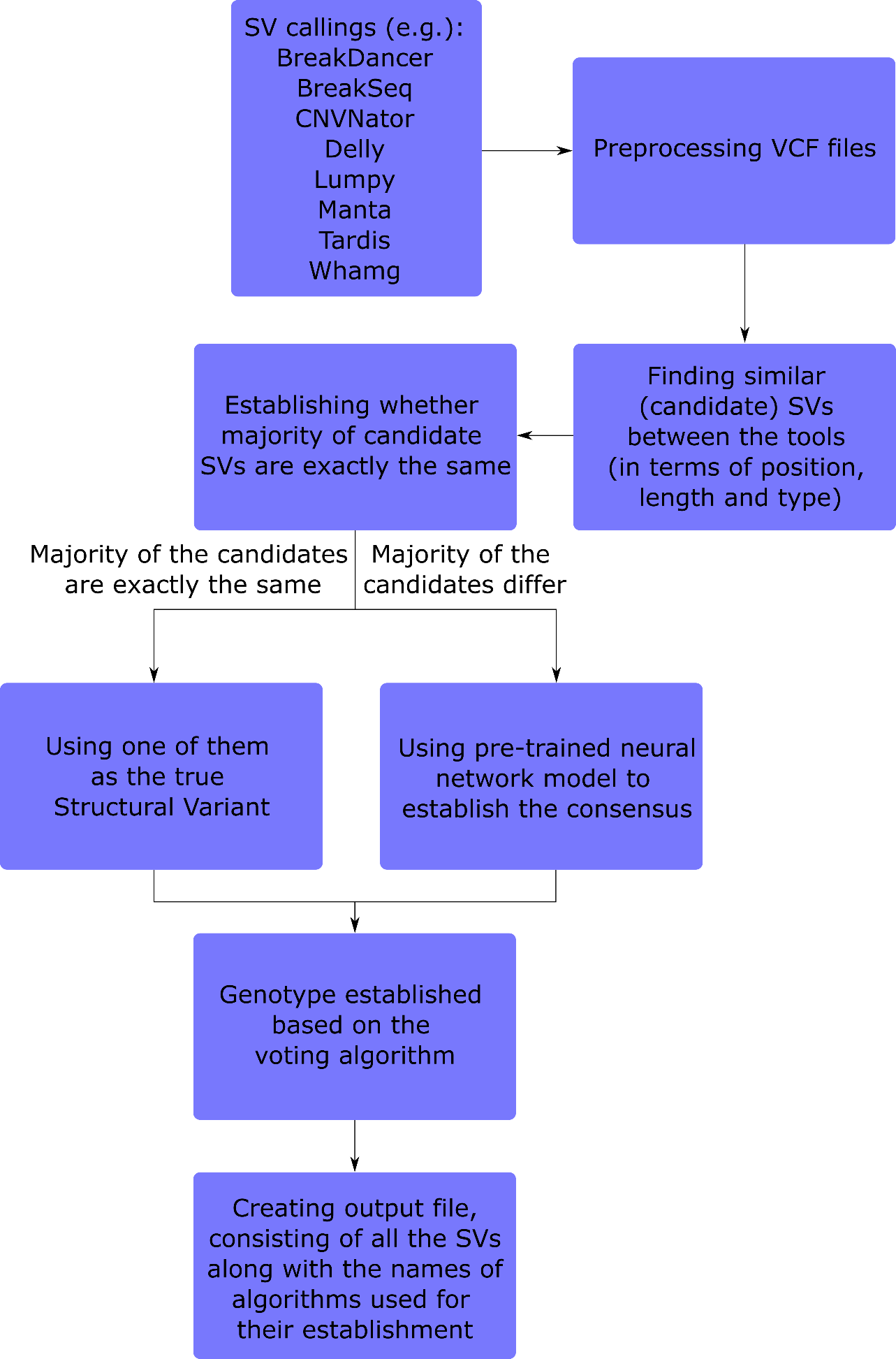


**Supplementary Figure 2.** ConsensuSV-core workflow.


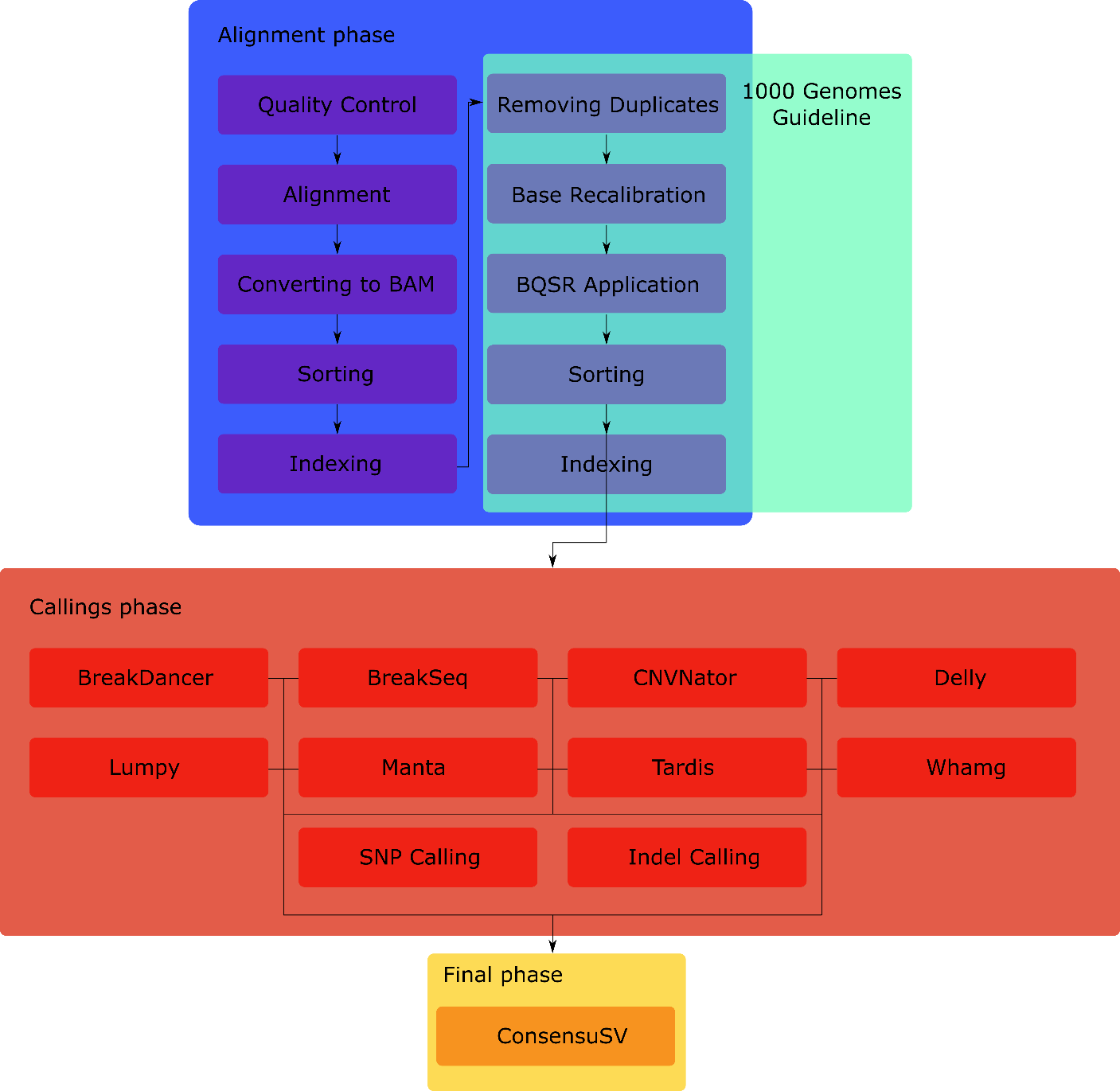


**Supplementary Figure 3.** ConsensuSV-pipeline workflow.

## Deep learning model of breakpoint positions

The *ConsensuSV-core* algorithm uses the deep learning architecture with input of *n* tools, two hidden layers – one with *2n* neurons, and the other with *n* neurons. The output of the network is the breakpoint positions. The algorithm by default uses pre-trained model that was trained using SVs obtained from the 8 previously mentioned tools (as ground truth for establishing breakpoints, the aforementioned gold-standard Illumina callset (Chaisson et al., 2019) was utilized – two families were used for training, and one for validation of the network). The simplified DL network for the default model created, and used in ConsensuSV-Pipeline (incorporating aforementioned 8 tools) can be seen in the **Supplementary Figure 4** (LeNail, 2019).


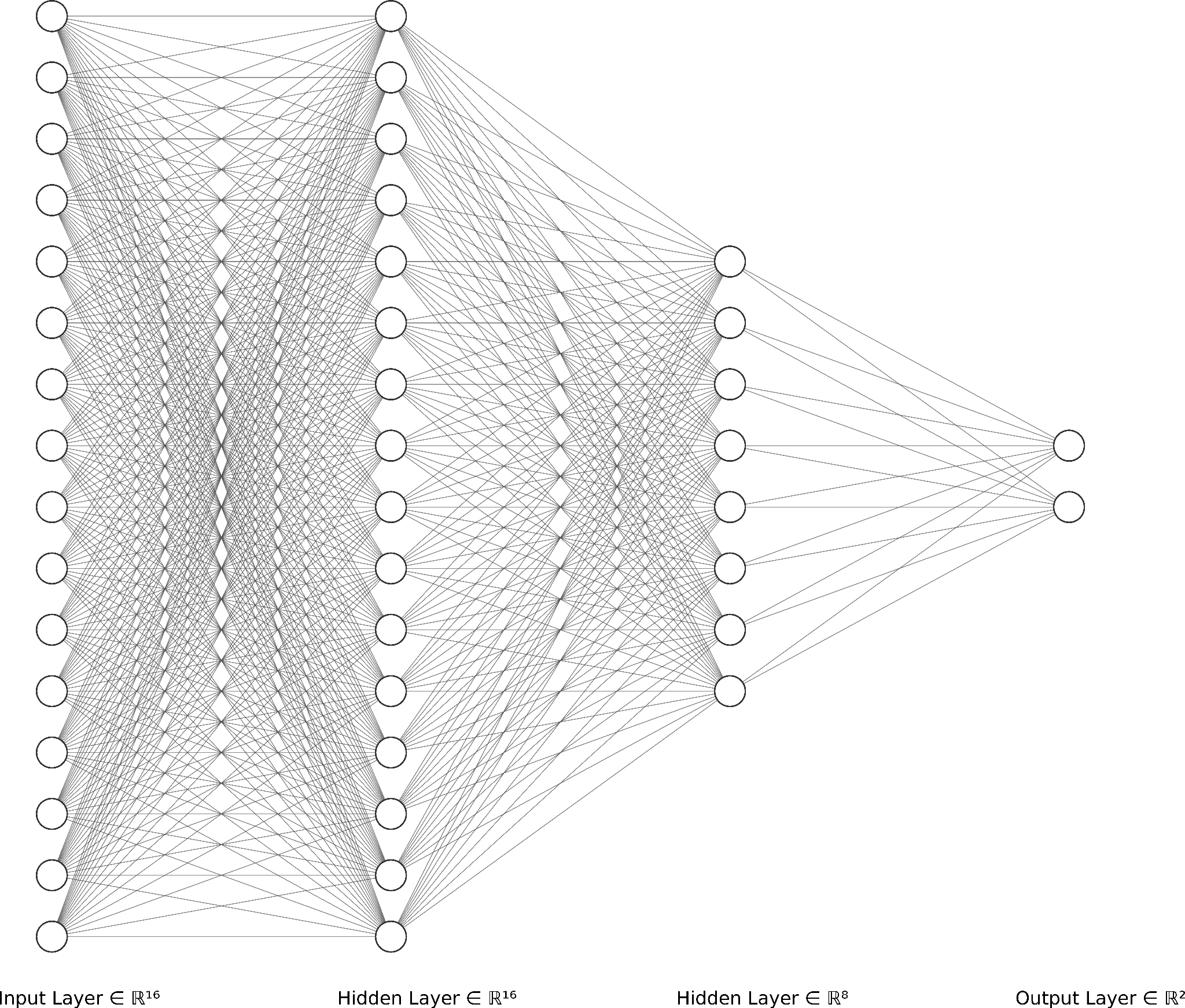


**Supplementary Figure 4.** Simplified deep learning architecture used in ConsensuSV-Core used in ConsensuSV-Pipeline.

## Parameters of minimal overlap

The suggested parameters are based mostly on the software that is used for SV calling. Since deletions are detected by all 8 algorithms, the suggested threshold is 3. For the remaining types, we suggest using the threshold of 2, since they are detected by 5 or 6 SV tools. We have made an exception for duplications, since along deletions, it’s easiest type of SV to detect, hence the SV callers detect them more frequently. The suggested parameters can be seen in the **Supplementary Table 3**.

| Type of variant | Suggested parameter |
| --- | --- |
| DEL (Deletion) | 3 |
| INS (Insertion) | 2 |
| DUP (Duplication) | 3 |
| INV (Inversion) | 2 |
| TRA (Translocation) | 2 |
| UNK (Unknown) | 3 |

**Supplementary Table 3.** Suggested parameters for ConsensuSV algorithm.

## Genotyping

We have decided to use the majority voting, because the vast majority of the tools agree on the genotype. We use 6 samples (HG00512, HG00513, HG00514, HG00731, HG00732, HG00733) for the training which account to 23291 SVs. Out of those, only 486 (2.1%) have genotype that is different across the tools used for establishment of the consensus. Unfortunately, we consider 486 examples to be insufficient for the proper teaching of any machine learning algorithm, as it would probably overfit greatly. Thus, we believe, that the voting algorithm is the best fit, since it correctly assigns genotype to 97.9% of the SVs (in case the tools report the genotype uniformly) and remaining 2.1% is based on the highest support of the tools that detect the variant.

# Meta-caller analysis

The benchmark study was conducted using NYGC-sequenced samples (Byrska-Bishop *et al.*, 2021): HG00512, HG00513, HG00514, HG00731, HG00732, HG00733, NA19238, NA19239, NA19240 sample available also in the 1000 Genomes project. The files used for analysis are shown in the **Supplementary Table 4**.

| HG00512 | |  | HG00733 | |
| --- | --- | --- | --- | --- |
| Forward | Reverse |  | Forward | Reverse |
| ERR3988780_1.fastq.gz | ERR3988780_2.fastq.gz |  | ERR3988823_1.fastq.gz | ERR3988823_2.fastq.gz |
|  | |  |  | |
| HG00513 | |  | NA19238 | |
| Forward | Reverse |  | Forward | Reverse |
| ERR3241684_1.fastq.gz | ERR3241684_2.fastq.gz |  | ERR3239453_1.fastq.gz | ERR3239453_2.fastq.gz |
| ERR4146610_1.fastq.gz | ERR4146610_2.fastq.gz |  | ERR4463894_1.fastq.gz | ERR4463894_2.fastq.gz |
| ERR4146611_1.fastq.gz | ERR4146611_2.fastq.gz |  | ERR4463895_1.fastq.gz | ERR4463895_2.fastq.gz |
| ERR4146612_1.fastq.gz | ERR4146612_2.fastq.gz |  | ERR4463896_1.fastq.gz | ERR4463896_2.fastq.gz |
| ERR4146613_1.fastq.gz | ERR4146613_2.fastq.gz |  | ERR4463897_1.fastq.gz | ERR4463897_2.fastq.gz |
| ERR4146614_1.fastq.gz | ERR4146614_2.fastq.gz |  | ERR4463898_1.fastq.gz | ERR4463898_2.fastq.gz |
| ERR4146615_1.fastq.gz | ERR4146615_2.fastq.gz |  | ERR4463899_1.fastq.gz | ERR4463899_2.fastq.gz |
| ERR4146616_1.fastq.gz | ERR4146616_2.fastq.gz |  | ERR4463900_1.fastq.gz | ERR4463900_2.fastq.gz |
| ERR4146617_1.fastq.gz | ERR4146617_2.fastq.gz |  | ERR4463901_1.fastq.gz | ERR4463901_2.fastq.gz |
| ERR4146618_1.fastq.gz | ERR4146618_2.fastq.gz |  | ERR4463902_1.fastq.gz | ERR4463902_2.fastq.gz |
| ERR4146619_1.fastq.gz | ERR4146619_2.fastq.gz |  | ERR4463903_1.fastq.gz | ERR4463903_2.fastq.gz |
| ERR4146620_1.fastq.gz | ERR4146620_2.fastq.gz |  | ERR4463904_1.fastq.gz | ERR4463904_2.fastq.gz |
| ERR4146621_1.fastq.gz | ERR4146621_2.fastq.gz |  | ERR4463905_1.fastq.gz | ERR4463905_2.fastq.gz |
|  | |  | ERR4463906_1.fastq.gz | ERR4463906_2.fastq.gz |
| HG00514 | |  |  | |
| Forward | Reverse |  | NA19239 | |
| ERR3988781_1.fastq.gz | ERR3988781_2.fastq.gz |  | Forward | Reverse |
|  | |  | ERR3239454_1.fastq.gz | ERR3239454_2.fastq.gz |
| HG00731 | |  | ERR3960750_1.fastq.gz | ERR3960750_2.fastq.gz |
| Forward | Reverse |  | ERR3960751_1.fastq.gz | ERR3960751_2.fastq.gz |
| ERR3241754_1.fastq.gz | ERR3241754_2.fastq.gz |  | ERR3960752_1.fastq.gz | ERR3960752_2.fastq.gz |
| ERR4146524_1.fastq.gz | ERR4146524_2.fastq.gz |  | ERR3960753_1.fastq.gz | ERR3960753_2.fastq.gz |
| ERR4146525_1.fastq.gz | ERR4146525_2.fastq.gz |  | ERR3960754_1.fastq.gz | ERR3960754_2.fastq.gz |
| ERR4146526_1.fastq.gz | ERR4146526_2.fastq.gz |  | ERR3960755_1.fastq.gz | ERR3960755_2.fastq.gz |
| ERR4146527_1.fastq.gz | ERR4146527_2.fastq.gz |  | ERR3960756_1.fastq.gz | ERR3960756_2.fastq.gz |
| ERR4146528_1.fastq.gz | ERR4146528_2.fastq.gz |  | ERR3960757_1.fastq.gz | ERR3960757_2.fastq.gz |
| ERR4146529_1.fastq.gz | ERR4146529_2.fastq.gz |  | ERR3960758_1.fastq.gz | ERR3960758_2.fastq.gz |
| ERR4146530_1.fastq.gz | ERR4146530_2.fastq.gz |  | ERR3960759_1.fastq.gz | ERR3960759_2.fastq.gz |
| ERR4146531_1.fastq.gz | ERR4146531_2.fastq.gz |  | ERR3960760_1.fastq.gz | ERR3960760_2.fastq.gz |
| ERR4146532_1.fastq.gz | ERR4146532_2.fastq.gz |  | ERR3960761_1.fastq.gz | ERR3960761_2.fastq.gz |
| ERR4146533_1.fastq.gz | ERR4146533_2.fastq.gz |  | ERR4983712_1.fastq.gz | ERR4983712_2.fastq.gz |
| ERR4146534_1.fastq.gz | ERR4146534_2.fastq.gz |  | ERR4983713_1.fastq.gz | ERR4983713_2.fastq.gz |
| ERR4146535_1.fastq.gz | ERR4146535_2.fastq.gz |  | ERR4983714_1.fastq.gz | ERR4983714_2.fastq.gz |
|  | |  | ERR4983715_1.fastq.gz | ERR4983715_2.fastq.gz |
| HG00732 | |  | ERR4983716_1.fastq.gz | ERR4983716_2.fastq.gz |
| Forward | Reverse |  | ERR4983717_1.fastq.gz | ERR4983717_2.fastq.gz |
| ERR3241755_1.fastq.gz | ERR3241755_2.fastq.gz |  | ERR4983718_1.fastq.gz | ERR4983718_2.fastq.gz |
| ERR4464635_1.fastq.gz | ERR4464635_2.fastq.gz |  | ERR4983719_1.fastq.gz | ERR4983719_2.fastq.gz |
| ERR4464636_1.fastq.gz | ERR4464636_2.fastq.gz |  | ERR4983720_1.fastq.gz | ERR4983720_2.fastq.gz |
| ERR4464637_1.fastq.gz | ERR4464637_2.fastq.gz |  | ERR4983721_1.fastq.gz | ERR4983721_2.fastq.gz |
| ERR4464638_1.fastq.gz | ERR4464638_2.fastq.gz |  | ERR4983722_1.fastq.gz | ERR4983722_2.fastq.gz |
| ERR4464639_1.fastq.gz | ERR4464639_2.fastq.gz |  | ERR4983723_1.fastq.gz | ERR4983723_2.fastq.gz |
| ERR4464640_1.fastq.gz | ERR4464640_2.fastq.gz |  |  | |
| ERR4464641_1.fastq.gz | ERR4464641_2.fastq.gz |  | NA19240 | |
| ERR4464642_1.fastq.gz | ERR4464642_2.fastq.gz |  | Forward | Reverse |
| ERR4464643_1.fastq.gz | ERR4464643_2.fastq.gz |  | ERR3989410_1.fastq.gz | ERR3989410_2.fastq.gz |
| ERR4464644_1.fastq.gz | ERR4464644_2.fastq.gz |  |  | |
| ERR4464645_1.fastq.gz | ERR4464645_2.fastq.gz |  |  |  |
| ERR4464646_1.fastq.gz | ERR4464646_2.fastq.gz |  |  |  |

**Supplementary Table 4.** Files used in the analysis.

## ConsensuSV-pipeline

The ConsensuSV-pipeline run was completed using the following command:

*python run_consensusv.py RunCSVFile --csv-file /data/consensusv_samples.csv --workers 4 --working-dir /working_dir/*

## MetaSV

The alignment files used for the processing of MetaSV (Mohiyuddin *et al.*, 2015) were prepared according to the HGSV recommendations (Chaisson *et al.*, 2019).

Breakdancer calls were done using the following commands:

metasv_working/breakdancer/perl/bam2cfg.pl HG00731/HG00731_preprocessed.bam > breakdancer_outputs/breakdancer_HG00731.cfg

metasv_working/breakdancer/bin/breakdancer-max breakdancer_outputs/breakdancer_HG00731.cfg > breakdancer_outputs/breakdancer_HG00731.out

CNVNator calls were done using the following commands:

metasv_working/CNVnator-master/cnvnator -root cnvnator_outputs/root_HG00731 -tree HG00731/HG00731_preprocessed.bam -chrom $(seq 1 22) X Y

metasv_working/CNVnator-master/cnvnator -root cnvnator_outputs/root_HG00731 -his 1000 -fasta GRCh38_full_analysis_set_plus_decoy_hla.fa

metasv_working/CNVnator-master/cnvnator -root cnvnator_outputs/root_HG00731 -stat 1000

metasv_working/CNVnator-master/cnvnator -root cnvnator_outputs/root_HG00731 -partition 1000

metasv_working/CNVnator-master/cnvnator -root cnvnator_outputs/root_HG00731 -call 1000 > cnvnator_outputs/cnvnator_HG00731.call

BreakSeq2 calls were done using the following commands:

run_breakseq2.py --reference GRCh38_full_analysis_set_plus_decoy_hla.fa --bams HG00731/HG00731_preprocessed.bam --work breakseq_outputs/breakseq_HG00731 --bwa metasv_working/bwa-0.7.17/bwa --samtools metasv_working/samtools-0.1.19/samtools --bplib_gff metasv_working/breakseq2_bplib_20150129_chr.gff --nthreads 4 --sample HG00731 --chromosomes chr1 chr2 chr3 chr4 chr5 chr6 chr7 chr8 chr9 chr10 chr11 chr12 chr13 chr14 chr15 chr16 chr17 chr18 chr19 chr20 chr21 chr22 chrX chrY chrM

Pindel calls were done using the following commands:

pindel/pindel -f GRCh38_full_analysis_set_plus_decoy_hla.fa -i metasv_working/HG00731_bam -o metasv_working/pindel_outputs/HG00731_pindel/ -w 100 -x 5 -T 16 --include pindel_include_chr.bed --exclude pindel-centromere-exclude.bed

The final merge using MetaSV was performed using the following command:

*run_metasv.py --reference /mnt/raid/GRCh38_full_analysis_set_plus_decoy_hla.fa --breakdancer_native breakdancer_outputs/breakdancer_HG00731.out --breakseq_native breakseq_outputs/breakseq_HG00731/breakseq.gff --cnvnator_native cnvnator_outputs/cnvnator_HG00731.call --pindel_native pindel_outputs/HG00731_pindel/_D pindel_outputs/HG00731_pindel/_LI pindel_outputs/HG00731_pindel/_SI pindel_outputs/HG00731_pindel/_TD pindel_outputs/HG00731_pindel/_INV --sample HG00731 --bam /mnt/raid/consensusv_paper/files_new_consensusv/pipeline/HG00731/HG00731_preprocessed.bam --spades SPAdes-3.15.3-Linux/bin/spades.py --age AGE/age_align --num_threads 8 --workdir metasv_working_dir/HG00731/ --outdir metasv_outputs/HG00731/ --min_support_ins 2 --max_ins_intervals 500000*

Only the Structural Variants passing the quality control were taken into the comparison.

## FusorSV

The input files were first merged, then the calls were done using following commands from FusorSV (Becker *et al.*, 2018):

*docker run -v fusorsv_working/:/data/ timothyjamesbecker/sve /software/SVE/scripts/prepare_bam.py -r /data/ref/human_g1k_v37/human_g1k_v37.fa -f /data/HG00512_R1.fastq,/data/HG00512_R2.fastq -o /data/fusor_HG00512/bams/ --cpus 4 --threads 1 --mem 4*

*docker run -it -v fusorsv_working/:/data/ timothyjamesbecker/sve /software/SVE/scripts/auto.py -r /data/ref/human_g1k_v37/human_g1k_v37.fa -b /data/fusor_HG00512/bams/HG00512_R.bam -o /data/fusor_HG00512/ --cpus 4 --threads 1 --mem 4*

Then, the output was liftovered to hg38 using crossmaping script (<https://github.com/timothyjamesbecker/FusorSV/blob/master/fusorsv/crossmap.py>).

# Benchmark study

## Comparisons using SVBench

For the comparisons of the precision of the callers, we have used SVBench (Cleal, 2020). We have used the charts showing precision vs. true positives (TP), and we have split the set based on the SVLEN – into the following size ranges:

- [0, 100)
- [100, 200)
- [200, 500)
- [500, 1000)
- [1000, 2000)
- [2000, 5000)
- [5000, 10000),
- [10000, 20000)
- [20000, 260000000)

The results can be seen in the **Supplementary Figure 5**.


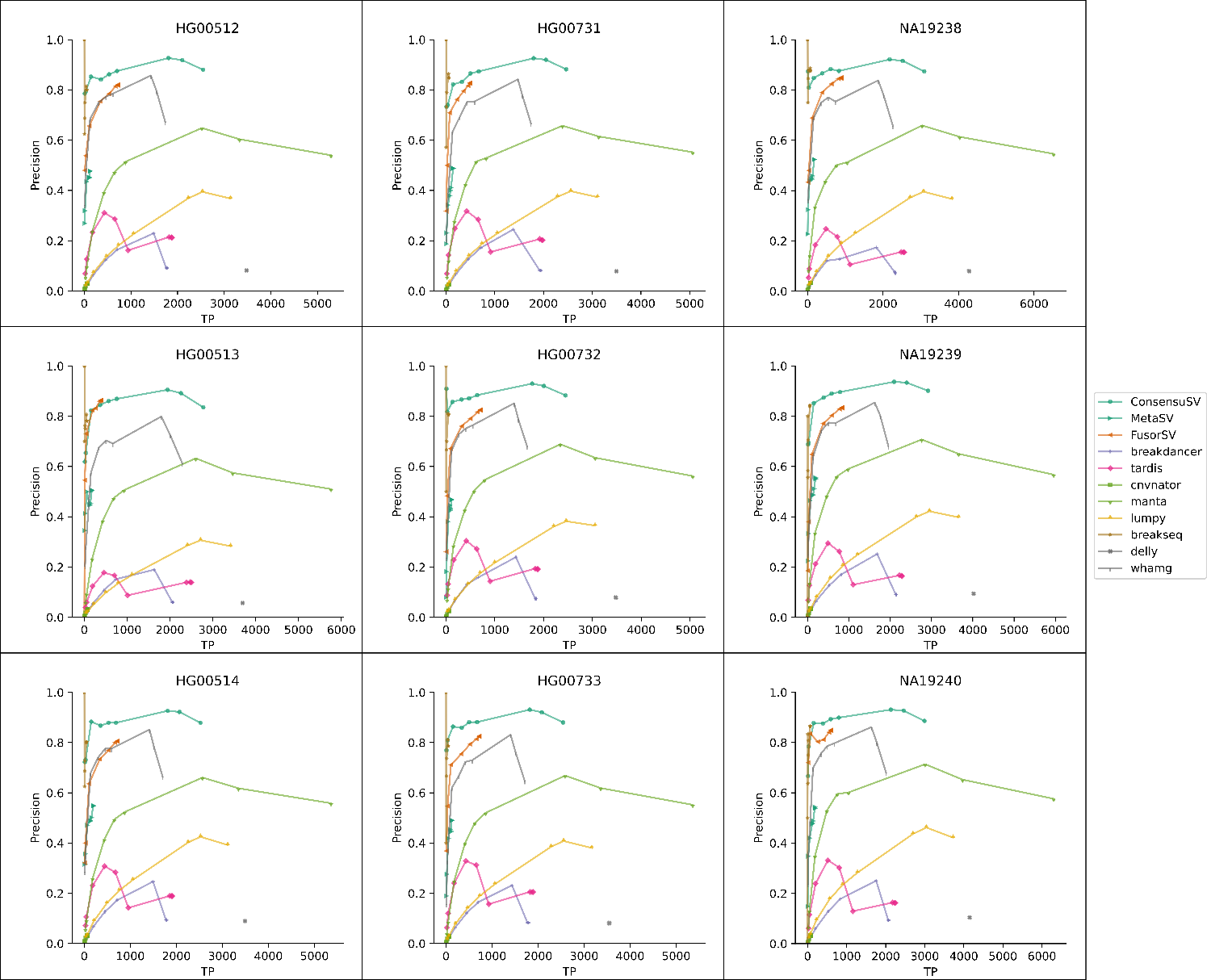


**Supplementary Figure 5.** Comparisons from SVBench – precision vs. true positives (TP).

## Comparisons using Venn diagrams

The comparisons between the output VCF files were done only on the breakpoints. The following command (Quinlan and Hall, 2010) was used:

*bedtools intersect -wa -header -f 0.8 -r -a tool_1.vcf -b tool_2.vcf > comparison.vcf*

The command compares two files, and takes only the breakpoints that are 80% similar between each other (e.g. variant A has to cover 80% of variant B, and variant B has to cover 80% of variant A to be considered as similar). Based on that, we have created Venn diagrams for each of the sample. We used those diagrams to create **Figure 1B**. The results are presented in the **Supplementary Figure 6**.


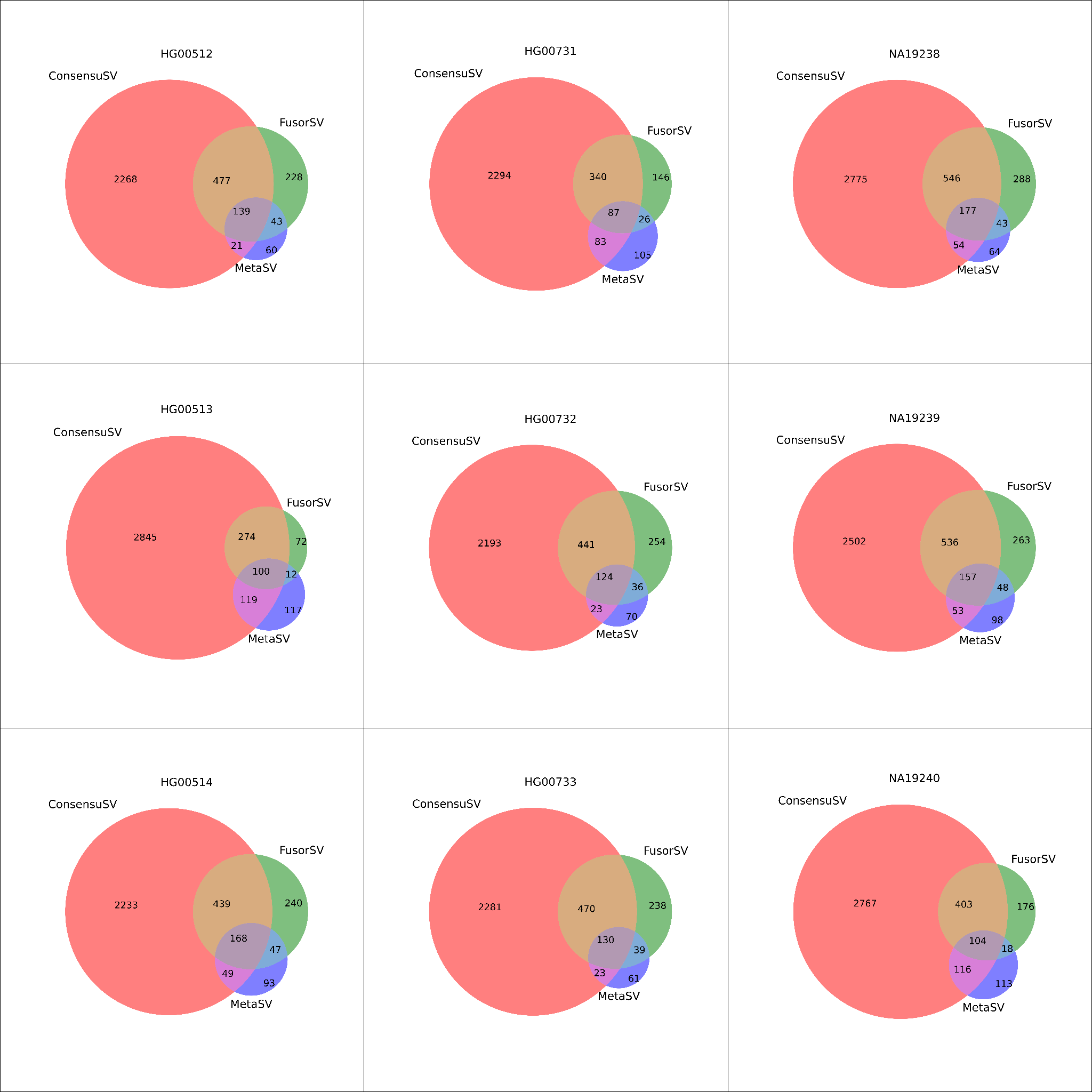


**Supplementary Figure 6.** Common SVs detected by ConsensuSV, FusorSV, and MetaSV.

# References

Abyzov,A. *et al.* (2015) Analysis of deletion breakpoints from 1,092 humans reveals details of mutation mechanisms. *Nat. Commun.*, **6**, 7256.

Abyzov,A. *et al.* (2011) CNVnator: an approach to discover, genotype, and characterize typical and atypical CNVs from family and population genome sequencing. *Genome Res.*, **21**, 974–984.

Auton,A. *et al.* (2015) A global reference for human genetic variation. *Nature*, **526**, 68–74.

Becker,T. *et al.* (2018) FusorSV: an algorithm for optimally combining data from multiple structural variation detection methods. *Genome Biol.*, **19**, 38.

Byrska-Bishop,M. *et al.* (2021) High coverage whole genome sequencing of the expanded 1000 Genomes Project cohort including 602 trios. *bioRxiv*, 2021.02.06.430068.

Chaisson,M.J.P. *et al.* (2019) Multi-platform discovery of haplotype-resolved structural variation in human genomes. *Nat. Commun.*, **10**, 1784.

Chen,K. *et al.* (2009) BreakDancer: an algorithm for high-resolution mapping of genomic structural variation. *Nat. Methods*, **6**, 677–681.

Chen,X. *et al.* (2016) Manta: rapid detection of structural variants and indels for germline and cancer sequencing applications. *Bioinformatics*, **32**, 1220–1222.

Cleal,K. (2020) kcleal/svbench: A python library for benchmarking structural variant calls against a reference set.

Kosugi,S. *et al.* (2019) Comprehensive evaluation of structural variation detection algorithms for whole genome sequencing. *Genome Biol.*, **20**, 117.

Kronenberg,Z.N. *et al.* (2015) Wham: Identifying Structural Variants of Biological Consequence. *PLoS Comput. Biol.*, **11**, e1004572–e1004572.

Layer,R.M. *et al.* (2014) LUMPY: a probabilistic framework for structural variant discovery. *Genome Biol.*, **15**, R84.

LeNail,A. (2019) NN-SVG: Publication-Ready Neural Network Architecture Schematics. *J. Open Source Softw.*, **4**, 747.

Mohiyuddin,M. *et al.* (2015) MetaSV: an accurate and integrative structural-variant caller for next generation sequencing. *Bioinformatics*, **31**, 2741–2744.

Quinlan,A.R. and Hall,I.M. (2010) BEDTools: a flexible suite of utilities for comparing genomic features. *Bioinformatics*, **26**, 841–842.

Rausch,T. *et al.* (2012) DELLY: structural variant discovery by integrated paired-end and split-read analysis. *Bioinformatics*, **28**, i333–i339.

Soylev,A. *et al.* (2019) Discovery of tandem and interspersed segmental duplications using high-throughput sequencing. *Bioinformatics*, **35**, 3923–3930.

Soylev,A. *et al.* (2017) Toolkit for automated and rapid discovery of structural variants. *Methods*, **129**, 3–7.
